# Supplementary material for: Polyunsaturated fatty acids inhibit a pentameric ligand-gated ion channel through one of two binding sites
Source: eLife. 2022 Jan 4;11:e74306. doi: 10.7554/eLife.74306 (PMC8786314; doi:10.7554/eLife.74306)
Supplement: Figure 4—source data 3. [file elife-74306-fig4-data3.docx]

DHA-PA-Linear-Mixed-Effects-Modeling-rev2.R

# Dependent Variable = Response
# Fixed Effect 1 = DHA
# Fixed Effect 2 = PA
# Fixed Effect 1 = Cysteamine
# Random Effect 2 = Replicate (Rep)

# Load packages
library(optimx)
library(dfoptim)
library(nloptr)
library(merTools)

library(glmmTMB)
library(brms)

library(modelr)
library(webshot)
library(magick)

library(afex)

library(tidyverse)

library(lme4)
library(lmerTest)

library(readxl)

# Subset DHA_Response_data and PA_Only_Response_data
DHA_Only_Response_data <- filter(Response_data, PA %in% 0)
DHA_Only_Response_subset <- dplyr::select(DHA_Only_Response_data, Rep, Cysteamine, DHA, Response)
view(DHA_Only_Response_subset)

PA_Only_Response_data <- filter(Response_data, DHA %in% 0)
PA_Only_Response_subset <- dplyr::select(PA_Only_Response_data, Rep, Cysteamine, PA, Response)
view(PA_Only_Response_subset)

# Testing for an effect of DHA or PA on Response
# Build a full model
DHA_Only_Response_subset %>%
 mutate(Cysteamine = factor(Cysteamine, levels = c("no_cys", "cys"), labels = c("0 mM Cys", "30 mM Cys"))) -> DHA_Only_Response_subset

DHA_Only_Response_subset.mod <- lmer(Response ~ Cysteamine + DHA + Cysteamine:DHA +(1|Rep), data = DHA_Only_Response_subset)

# View DHA summary output
summary(DHA_Only_Response_subset.mod)

## Linear mixed model fit by REML. t-tests use Satterthwaite's method [
## lmerModLmerTest]
## Formula: Response ~ Cysteamine + DHA + Cysteamine:DHA + (1 | Rep)
## Data: DHA_Only_Response_subset
##
## REML criterion at convergence: -31
##
## Scaled residuals:
## Min 1Q Median 3Q Max
## -1.3186 -0.2660 -0.1192 0.1920 2.6536
##
## Random effects:
## Groups Name Variance Std.Dev.
## Rep (Intercept) 0.002452 0.04952
## Residual 0.002348 0.04846
## Number of obs: 21, groups: Rep, 3
##
## Fixed effects:
## Estimate Std. Error df t value Pr(>|t|)
## (Intercept) 0.071531 0.037103 4.257230 1.928 0.1218
## Cysteamine30 mM Cys 0.017680 0.029755 15.000000 0.594 0.5612
## DHA 0.001858 0.001295 15.000000 1.434 0.1720
## Cysteamine30 mM Cys:DHA -0.004667 0.001727 15.000000 -2.703 0.0164 *
## ---
## Signif. codes: 0 '***' 0.001 '**' 0.01 '*' 0.05 '.' 0.1 ' ' 1
##
## Correlation of Fixed Effects:
## (Intr) Cy30mMC DHA
## Cystmn30mMC -0.506
## DHA -0.465 0.580
## Cy30mMC:DHA 0.349 -0.689 -0.750

sjPlot::tab_model(DHA_Only_Response_subset.mod, title= "DHA Response Summary Table", digits=4, digits.p = 3, digits.rsq = 3, digits.re = 4, file = "DHA Response Summary Table.html", use.viewer = TRUE)

DHA_Only_Response_subset %>%
 group_by(DHA, Cysteamine) %>%
 summarize(Response = mean(Response)) %>%
 ggplot(aes(DHA, Response, color = Cysteamine)) +
 geom_smooth(method = 'lm', se = FALSE) + ggtitle("DHA Concentration Response") + theme(plot.title = element_text(hjust= 0.5)) +theme(legend.position = "bottom") + xlab("DHA (uM)")

## `summarise()` has grouped output by 'DHA'. You can override using the `.groups` argument.

## `geom_smooth()` using formula 'y ~ x'

PA_Only_Response_subset %>%
 mutate(Cysteamine = factor(Cysteamine, levels = c("no_cys", "cys"), labels = c("0 mM Cys", "30 mM Cys"))) -> PA_Only_Response_subset

PA_Only_Response_subset.mod <- lmer(Response ~ Cysteamine + PA + Cysteamine:PA +(1|Rep), data = PA_Only_Response_subset)

## fixed-effect model matrix is rank deficient so dropping 1 column / coefficient

# View PA summary output
summary(PA_Only_Response_subset.mod)

## Linear mixed model fit by REML. t-tests use Satterthwaite's method [
## lmerModLmerTest]
## Formula: Response ~ Cysteamine + PA + Cysteamine:PA + (1 | Rep)
## Data: PA_Only_Response_subset
##
## REML criterion at convergence: -45.5
##
## Scaled residuals:
## Min 1Q Median 3Q Max
## -1.41722 -0.30194 -0.01592 0.28212 1.43314
##
## Random effects:
## Groups Name Variance Std.Dev.
## Rep (Intercept) 0.0029561 0.05437
## Residual 0.0002676 0.01636
## Number of obs: 15, groups: Rep, 3
##
## Fixed effects:
## Estimate Std. Error df t value Pr(>|t|)
## (Intercept) 0.0753331 0.0327803 2.2922749 2.298 0.132
## Cysteamine30 mM Cys 0.0159280 0.0112403 10.0000000 1.417 0.187
## PA -0.0004599 0.0003855 10.0000000 -1.193 0.260
##
## Correlation of Fixed Effects:
## (Intr) C30mMC
## Cystmn30mMC -0.242
## PA 0.000 -0.343
## fit warnings:
## fixed-effect model matrix is rank deficient so dropping 1 column / coefficient

PA_Only_Response_subset %>%
 group_by(PA, Cysteamine) %>%
 summarize(Response = mean(Response)) %>%
 ggplot(aes(PA, Response, color = Cysteamine)) + geom_smooth(method = 'lm', se = FALSE) + ggtitle("PA Concentration Response") + theme(plot.title = element_text(hjust= 0.5)) +theme(legend.position = "bottom") + xlab("PA (uM)")

## `summarise()` has grouped output by 'PA'. You can override using the `.groups` argument.
## `geom_smooth()` using formula 'y ~ x'

# Box Plots
# subset the DHA and PA Only subsets for Cysteamine
## No cys
DHA_Only_Response_box_no_cys <- filter(DHA_Only_Response_data, Cysteamine %in% 'no_cys')
DHA_Only_Response_box_no_cys_1 <- dplyr::select(DHA_Only_Response_box_no_cys, Rep, Cysteamine, DHA, Response)
view(DHA_Only_Response_box_no_cys_1)

PA_Only_Response_box_no_cys <- filter(PA_Only_Response_data, Cysteamine %in% 'no_cys')
PA_Only_Response_box_no_cys_1 <- dplyr::select(PA_Only_Response_box_no_cys, Rep, Cysteamine, PA, Response)
view(PA_Only_Response_box_no_cys_1)

## cys
DHA_Only_Response_box_cys <- filter(DHA_Only_Response_data, Cysteamine %in% 'cys')
DHA_Only_Response_box_cys_2 <- dplyr::select(DHA_Only_Response_box_cys, Rep, Cysteamine, DHA, Response)
view(DHA_Only_Response_box_cys_2)

PA_Only_Response_box_cys <- filter(PA_Only_Response_data, Cysteamine %in% 'cys')
PA_Only_Response_box_cys_2 <- dplyr::select(PA_Only_Response_box_cys, Rep, Cysteamine, PA, Response)
view(PA_Only_Response_box_cys_2)

ggplot(DHA_Only_Response_box_no_cys_1, aes(x=DHA, y=Response)) + aes(group = DHA) + geom_boxplot(outlier.colour="red", outlier.shape=8, outlier.size=4) + expand_limits(x=c(0,30), y=c(0, 0.3)) +theme(legend.position="bottom",legend.direction="horizontal") +ggtitle("DHA Concentration Response + 0 mM Cysteamine") +theme(plot.title = element_text(hjust= 0.5)) + xlab("DHA (uM)")

ggplot(PA_Only_Response_box_no_cys_1, aes(x=PA, y=Response)) + aes(group = PA) + geom_boxplot(outlier.colour="red", outlier.shape=8, outlier.size=4) + expand_limits(x=c(0,30), y=c(0, 0.3)) +theme(legend.position="bottom",legend.direction="horizontal") +ggtitle("PA Concentration Response + 0 mM Cysteamine") +theme(plot.title = element_text(hjust= 0.5)) + xlab("PA (uM)")

ggplot(DHA_Only_Response_box_cys_2, aes(x=DHA, y=Response)) + aes(group = DHA) + geom_boxplot(outlier.colour="red", outlier.shape=8, outlier.size=4)+ expand_limits(x=c(0,30), y=c(0, 0.3)) +theme(legend.position="bottom",legend.direction="horizontal") +ggtitle("DHA Concentration Response + 30 mM Cysteamine") +theme(plot.title = element_text(hjust= 0.5)) + xlab("DHA (uM)")

ggplot(PA_Only_Response_box_cys_2, aes(x=PA, y=Response)) + aes(group = PA) + geom_boxplot(outlier.colour="red", outlier.shape=8, outlier.size=4) + expand_limits(x=c(0,30), y=c(0, 0.3)) +theme(legend.position="bottom",legend.direction="horizontal") +ggtitle("PA Concentration Response + 30 mM Cysteamine") +theme(plot.title = element_text(hjust= 0.5)) + xlab("PA (uM)")
