# Supplementary material for: Polyunsaturated fatty acids inhibit a pentameric ligand-gated ion channel through one of two binding sites
Source: eLife. 2022 Jan 4;11:e74306. doi: 10.7554/eLife.74306 (PMC8786314; doi:10.7554/eLife.74306)
Supplement: Transparent reporting form [file elife-74306-transrepform1.docx]

***eLife’s* transparent reporting form**

We encourage authors to provide detailed information *within their submission* to facilitate the interpretation and replication of experiments. Authors can upload supporting documentation to indicate the use of appropriate reporting guidelines for health-related research (see [EQUATOR Network](http://www.equator-network.org/%20)), life science research (see the [BioSharing Information Resource](https://biosharing.org/)), or the [ARRIVE guidelines](http://www.plosbiology.org/article/info:doi/10.1371/journal.pbio.1000412) for reporting work involving animal research. Where applicable, authors should refer to any relevant reporting standards documents in this form.

If you have any questions, please consult our Journal Policies and/or contact us: [editorial@elifesciences.org](mailto:editorial@elifesciences.org).

**Sample-size estimation**

- You should state whether an appropriate sample size was computed when the study was being designed
- You should state the statistical method of sample size computation and any required assumptions
- If no explicit power analysis was used, you should describe how you decided what sample (replicate) size (number) to use

Please outline where this information can be found within the submission (e.g., sections or figure legends), or explain why this information doesn’t apply to your submission:

Sample sizes for each experiment and statistical method for analysis are indicated in each figure legend. No power analysis was performed. For most experiments, sample sizes of 3-6 were deemed sufficient because the data showed good precision and the presence or absence of significant differences was apparent in each case. For recordings of peak current/current density in Figure 6B, sample sizes (number of recordings) were increased to 12+ due to the expected high variability in peak current observed between independent excised patch-clamp recordings.

**Replicates**

- You should report how often each experiment was performed
- You should include a definition of biological versus technical replication
- The data obtained should be provided and sufficient information should be provided to indicate the number of independent biological and/or technical replicates
- If you encountered any outliers, you should describe how these were handled
- Criteria for exclusion/inclusion of data should be clearly stated
- High-throughput sequence data should be uploaded before submission, with a private link for reviewers provided (these are available from both GEO and ArrayExpress)

Please outline where this information can be found within the submission (e.g., sections or figure legends), or explain why this information doesn’t apply to your submission:

All data are biological replicates. Specifically, electrophysiology data were analyzed from independent excised patches from the same batch of giant proteoliposomes. Mass spectrometry data were obtained from independent photolabeling, trypsin digests, and LC-MS analyses, performed from the same batch of purified protein. No data were excluded as outliers.

**Statistical reporting**

- Statistical analysis methods should be described and justified
- Raw data should be presented in figures whenever informative to do so (typically when N per group is less than 10)
- For each experiment, you should identify the statistical tests used, exact values of N, definitions of center, methods of multiple test correction, and dispersion and precision measures (e.g., mean, median, SD, SEM, confidence intervals; and, for the major substantive results, a measure of effect size (e.g., Pearson's r, Cohen's d)
- Report exact p-values wherever possible alongside the summary statistics and 95% confidence intervals. These should be reported for all key questions and not only when the p-value is less than 0.05.

Please outline where this information can be found within the submission (e.g., sections or figure legends), or explain why this information doesn’t apply to your submission:

As indicated in the results section, all statistical analyses were performed using a one-way ANOVA with Tukey post hoc HSD test. This is appropriate in most cases where >2 independent conditions were compared for significant differences. In all figure legends, sample size, error bars (SEM), and p-values are indicated. Two conditions in Figure 6B were also analyzed using an unpaired T-test as indicated in the results section. This was performed to compare two conditions that belonged to the same batch of liposomes, providing additional information about how these conditions compare. A linear mixed effects model was used to analyze the photolabeling competition data from Figure 4C and Figure 4- figure supplement 5. The details of this analysis can be found in the figure legends and Figure 4- source data 3.

(For large datasets, or papers with a very large number of statistical tests, you may upload a single table file with tests, Ns, etc., with reference to sections in the manuscript.)

**Group allocation**

- Indicate how samples were allocated into experimental groups (in the case of clinical studies, please specify allocation to treatment method); if randomization was used, please also state if restricted randomization was applied
- Indicate if masking was used during group allocation, data collection and/or data analysis

Please outline where this information can be found within the submission (e.g., sections or figure legends), or explain why this information doesn’t apply to your submission:

Sample randomization and masking do not apply to this study.

**Additional data files (“source data”)**

- We encourage you to upload relevant additional data files, such as numerical data that are represented as a graph in a figure, or as a summary table
- Where provided, these should be in the most useful format, and they can be uploaded as “Source data” files linked to a main figure or table
- Include model definition files including the full list of parameters used
- Include code used for data analysis (e.g., R, MatLab)
- Avoid stating that data files are “available upon request”

Please indicate the figures or tables for which source data files have been provided:

Source data files are provided for Figure 4A and 4B (source data 1), and Figure 4C and Figure 4- figure supplement 5 (source data 2 and 3).
